# Precise pathogen detection and clinical characterization of bronchiectasis

**DOI:** 10.3389/fcimb.2025.1670925

**Published:** 2025-09-26

**Authors:** Qinghua Gao, Jiahao Xu, Yue Ma, Siji Zhou, Lei Zhang, Liping Chen, Yongning Yi, Ting Hou, Qiaoli Zhang, Jian He

**Affiliations:** ^1^ Department of Pulmonary and Critical Care Medicine, The Anning First People’s Hospital Affiliated to Kunming University of Science and Technology, Kunming, China; ^2^ Yunnan Taite Biotech, Kunming, China

**Keywords:** bronchiectasis, clinical characteristics, molecular diagnostic, *Haemophilus influenzae*, Pseudomonas aeruginosa

## Abstract

**Introduction:**

This study aims to evaluate the utility of molecular diagnostic techniques in identifying pathogens in bronchiectasis and to investigate the differences in clinical characteristics and pathogen distribution among patients with different microbial infections.

**Methods:**

This retrospective study collected and analyzed clinical data and lower respiratory tract pathogen detection results from 410 patients with bronchiectasis admitted to the Anning First People’s Hospital Affiliated to Kunming University of Science and Technology, between August 2020 and August 2024. By comparing molecular diagnostic methods with conventional culture, we assessed differences in pathogen detection rates and spectrum, evaluated the diagnostic performance of molecular techniques relative to traditional methodologies, and analyzed the clinical characteristics of bronchiectasis patients with different microbiological etiologies.

**Results:**

Compared with conventional microbiological testing (CMT), molecular diagnostics demonstrated significantly higher sensitivity, positive predictive value, and negative predictive value. The most frequently detected bacteria were *Haemophilus influenzae* (26.83%), *Pseudomonas aeruginosa* (14.88%), *Streptococcus pneumoniae* (13.17%), *Klebsiella pneumoniae* (9.02%), and *Staphylococcus aureus* (4.39%). Patients infected with *P. aeruginosa* had significantly lower body mass index (BMI) compared to those infected with *H. influenzae*, as well as more severe lung function impairment. Inflammatory markers, including white blood cell (WBC) count and C-reactive protein (CRP), were significantly higher in the *P. aeruginosa* group than in the *H. influenzae* group. In terms of pathogen detection, the conventional culture positivity rate was significantly higher in the *P. aeruginosa* group compared to the *H. influenzae* group, whereas the false-negative rate of culture was markedly higher in the *H. influenzae* group. Molecular diagnostics showed high true-positive rates in both groups, though slightly lower in the *P. aeruginosa* group than in the *H. influenzae* group. Furthermore, patients infected with *P. aeruginosa* had significantly higher rates of respiratory failure, cystic bronchiectasis, and oxygen therapy requirement compared to those infected with *H. influenzae*.

**Conclusion:**

The application of molecular diagnostic technology has significantly improved the detection rate of pathogens in patients with bronchiectasis, especially for fastidious bacteria and rare pathogens. This method can provide a more comprehensive understanding of the distribution of microorganisms and disease characteristics, shorten the diagnosis cycle, accurately guide anti-infection treatment decisions and assist in prognosis assessment.

## 1 Introduction

Bronchiectasis is a chronic and progressive respiratory disease characterized by irreversible bronchial dilatation, persistent inflammation, and recurrent infections (Xu et al., 2022). It is considered a highly heterogeneous disease with a variety of clinical manifestations, imaging findings, etiologies, and pathogenic features (Flume et al., 2018; Bedi et al., 2021; Aliberti et al., 2022). Its etiological diagnosis plays a crucial role in disease management. A core feature of its pathophysiology is the presence of chronic or recurrent infections, where the persistent presence of pathogens leads to repeated bronchial infections, impaired mucociliary clearance, and a vicious cycle of airway damage and inflammation (Polverino et al., 2017; Choi et al., 2024). Therefore, accurate and timely identification of pathogenic microorganisms is essential for guiding appropriate antimicrobial therapy, risk stratification, and long-term management of the disease. However, large-scale registry studies such as the BE-China study have provided valuable insights into the clinical and microbiological profile of bronchiectasis in China (Xu et al., 2025), but significant differences still exist between regions, and data from Yunnan are still scarce.

The microbiological diagnosis of bronchiectasis has long relied on culture (Guan et al., 2015; Aliberti et al., 2016). However, these methods have significant several limitations, including low sensitivity, prolonged turnaround times and particularly in the identification of fastidious and uncommon pathogens such as *Haemophilus influenzae*, *Moraxella catarrhalis*, *Mycobacterium tuberculosis*, *Non-tuberculous mycobacteria*, *Nocardia* sp*ecies*, and fungi (Chalmers et al., 2018; Duan et al., 2024; Zhao et al., 2024). These limitations contribute to uncertain microbiological diagnoses, inappropriate treatment strategies, and delays in clinical intervention.

Molecular diagnostic techniques, such as metagenomic next-generation sequencing (mNGS), targeted next-generation sequencing (tNGS), and multiplex quantitative PCR (qPCR), have significantly expanded the scope and sensitivity of pathogen detection (Ponsford et al., 2021; Duan et al., 2024; Hong et al., 2024; Chen et al., 2025). These techniques allow for rapid, sensitive, and comprehensive identification of a broad range of microorganisms directly from clinical specimens. Emerging evidence suggests that molecular methods not only improve diagnostic yield but may also offer prognostic value by identifying pathogen-specific clinical phenotypes (Zhang et al., 2023; Shen et al., 2024). However, there remains a need for real-world data to validate their utility in routine clinical settings and to clarify the relationship between microbial profiles and disease severity.

This study was a single-center, retrospective cohort analysis of 410 patients diagnosed with bronchiectasis. By comparing molecular diagnostic methods with conventional microbiological testing, we aimed to evaluate the etiological diagnostic performance of molecular techniques and describe the microbial spectrum of patients with bronchiectasis in the region. In addition, we analyzed the differences in clinical characteristics, severity, and prognosis among patients infected with the two most common bacterial pathogens, *P. aeruginosa* and *H. influenzae*.

## 2 Methods

### 2.1 Patients and study design

This retrospective observational study included clinical data from 410 patients diagnosed with bronchiectasis who were admitted to the Department of Respiratory and Critical Care Medicine at Anning First People’s Hospital Affiliated to Kunming University of Science and Technology between August 2020 and August 2024. The study was approved by the Ethics Committee of Anning First People’s Hospital (Approval No. 2023-077 (Science)-01). It was conducted in accordance with the ethical principles outlined in the Declaration of Helsinki, and all patient data were handled with strict confidentiality. As this was a retrospective study with anonymized data, informed consent was not required.

The diagnosis of bronchiectasis was based on high-resolution computed tomography (HRCT) findings showing bronchial dilatation in one or more pulmonary lobes, accompanied by clinical symptoms such as chronic cough, sputum production, and/or recurrent respiratory exacerbations (Polverino et al., 2017; Hill et al., 2019). Exclusion criteria included age under 18 years, traction bronchiectasis associated with interstitial lung disease, inability to provide clinical records, or absence of microbiological testing data.

### 2.2 Specimen collection and processing

For each patient, only the first BALF specimen obtained during the index hospitalization was included in the analysis, and no repeat samples were considered. All patients underwent bronchoalveolar lavage within 24–48 hours of hospital admission. The procedure was performed by experienced pulmonologists in accordance with the American Thoracic Society (ATS) and European Respiratory Society (ERS) clinical practice guideline for bronchoalveolar lavage standardization (Meyer et al., 2012). Briefly, bronchoalveolar lavage was performed in the most radiologically involved bronchopulmonary segment, with at least 100–150 mL of sterile saline instilled in aliquots of 20–50 mL. A minimum of 30% of the instilled volume was recovered to ensure adequate sampling.

For microbiological culture, bronchoalveolar lavage fluid (BALF) specimens were transported to the microbiology laboratory under refrigerated conditions (2–8°C) and inoculated onto culture media within 2 hours of collection. For molecular diagnostics (multiplex qPCR, tNGS, or mNGS), aliquots of the same BALF were stored at 4°C and delivered to the sequencing laboratory within 12 hours of collection. All specimens were handled promptly to minimize nucleic acid degradation and ensure consistency across patients.

### 2.3 Clinical data collection

Data collected from the medical record system included age, sex, smoking history, and body mass index (BMI). The comorbidities were categorized as follows: chronic obstructive pulmonary disease (COPD); asthma; other pulmonary diseases, including interstitial lung disease, pulmonary tuberculosis, pneumoconiosis and interstitial pulmonary fibrosis, etc.; cardiovascular and cerebrovascular diseases, such as congestive heart failure, atrial septal defect, and ventricular septal defect; immunosuppression; malignancies, defined as a confirmed diagnosis of cancer prior to hospital admission; metabolic disorders, such as diabetes mellitus; and aspiration risk, which included conditions such as cerebral infarction, cerebral hemorrhage, coma, epilepsy, prolonged bedridden status, hiatal hernia, and gastroesophageal reflux disease. Clinical variables included pulmonary function parameters [FEV_1_ and FEV_1_/FVC], inflammatory markers [white blood cell (WBC) count, neutrophil count, and C-reactive protein (CRP)], radiologic findings (presence of cystic bronchiectasis and mucus plugging), oxygen therapy status, respiratory failure, and history of acute exacerbation (AE). Disease severity was assessed using the Bronchiectasis Severity Index (BSI).

Microbiological data include conventional microbiological testing (CMT) and molecular diagnostic methods. Pathogen detection samples are collected from the lower respiratory tract (bronchoalveolar lavage fluid obtained by bronchoscopy). The classification of true positive, true negative, false positive, and false negative results was based on a composite clinical reference standard. This adjudication incorporated (i) clinical manifestations, (ii) radiological findings, (iii) results of conventional microbiological testing, (iv) molecular diagnostic results, and (v) the patient’s treatment response. The above process must be reviewed by two experienced bronchiectasis subgroup clinicians (ZSJ and MY). Any disagreement between the two physicians is resolved through in-depth discussion. If a consensus cannot be reached, it will be judged by another senior physician (ZQL) to ensure the consistency of clinical diagnosis.

This composite approach was adopted because no universally accepted gold standard exists for pathogen identification in bronchiectasis, and conventional culture alone is known to have low sensitivity and a high false-negative rate, particularly for fastidious organisms. Importantly, our adjudication strategy is consistent with recent expert consensus and practice guidelines for the application of mNGS and tNGS in respiratory infections, including the Chinese Thoracic Society consensus on clinical pathways of mNGS testing in lower respiratory tract infections and the Chinese Medical Association expert consensus on mNGS for infectious disease diagnosis (Subspecialty Group of Neonatologyet al., 2022; Chinese Thoracic Society, 2023; Kuang et al., 2024). These documents explicitly recommend that molecular diagnostic results should not be interpreted in isolation, but rather in conjunction with clinical features, imaging findings, and treatment response, in order to minimize misclassification and improve clinical relevance.

### 2.4 Pathogen detection

#### 2.4.1 Conventional microbiological testing

Routine microbiological culture and smear examination were performed on lower respiratory tract specimens collected from all patients. For patients with clinical suspected viral infection, a respiratory multiplex PCR panel was used to detect SARS-CoV-2, influenza A virus, influenza B virus, parainfluenza virus, adenovirus, respiratory syncytial virus (RSV), and human metapneumovirus (HMPV). For patients with suspected Aspergillus infection, bronchoalveolar lavage fluid galactomannan (GM) was tested. For patients with suspected tuberculosis, acid-fast staining and GeneXpert testing were performed.

#### 2.4.2 Metagenomic next-generation sequencing

The methods of mNGS were the same as that described in our previously published article (Gao et al., 2024), mNGS was performed on BALF. For RNA samples, reverse transcription was carried out prior to library preparation. Sequencing libraries were constructed through enzymatic fragmentation, end repair, adapter ligation, and index tagging, followed by quantification using real-time PCR. Libraries were then pooled and subjected to shotgun sequencing on the Illumina NextSeq platform, generating approximately 20 million 50-bp single-end reads per sample. Raw sequencing data underwent a standardized bioinformatics pipeline. To minimize host interference, sequencing reads were first mapped to the human reference genome (GRCh38.p13) using Burrows–Wheeler alignment (BWA), and all human-derived reads were removed. After low-quality and low-complexity reads were filtered out, the remaining reads were aligned against the NCBI nt and GenBank databases for microbial identification.

For reporting standards, microbial species were considered positive if (i) sequencing quality passed predefined thresholds (library concentration > 50 pM, Q20 > 85%, Q30 > 80%), and (ii) the corresponding negative control (NC) from the same sequencing batch did not contain the species, or the ratio of RPM in the sample to RPM in the NC exceeded 5. This empirically determined cutoff was applied to reduce background contamination and distinguish true positives from environmental or reagent-derived noise. All sequencing runs included negative controls to monitor potential contamination.

#### 2.4.3 Targeted next-generation sequencing

TNGS was used to detect predefined clinically relevant pathogens through a multiplex PCR-based approach. The panel covered 363 pathogens, including 231 bacteria, 65 viruses, 48 fungi, and 19 parasites. The assay was developed and performed by Hangzhou Matridx Biotechnology Co., Ltd. (Hangzhou, China). Sample processing and nucleic acid extraction from BALF were performed using the same protocol as for mNGS. Separate DNA and RNA workflows were established: RNA viruses were reverse transcribed into cDNA, and both genomic DNA and cDNA were subjected to multiplex PCR to enrich target sequences. Amplified products from the two reactions were then pooled, purified, and ligated with sequencing adapters and barcodes to construct the final sequencing library. Library quality control, pooling, DNB preparation, and sequencing were conducted similarly to mNGS, except that tNGS generated approximately 500,000 reads per sample. After sequencing, the reads were first aligned to the human reference genome (GRCh38.p13) to remove host-derived sequences. The remaining reads were then subjected to quality control to filter out short or non-specific fragments, and the resulting clean reads were aligned to reference databases for pathogen identification. Organism-level positivity was defined if specific pathogen reads exceeded empirically determined thresholds (≥ 10 reads or RPM(sample)/RPM(NC) > 5), and were absent or negligible in negative controls. Each run included no-template controls to monitor potential contamination. All samples and controls were processed in parallel within the same sequencing batch to minimize batch effects,

#### 2.4.4 Multiplex fluorescence quantitative PCR

BALF samples were collected and commissioned to the Precision Medicine Testing Laboratory of Kunming Medical University for nucleic acid testing of 36 respiratory-related pathogens (See **Appendix Table A1 for details of pathogens**). Referring to the instructions of the reagent supplier, viral DNA/RNA nucleic acid extraction reagent (Xi’an Tianlong, Suzhou) was used to extract nucleic acids from the samples, and then the amplification reaction system was prepared according to the instructions of the multiplexed fluorescence quantitative PCR assay reagent, and 5 μL of nucleic acid template was added to each reaction tube. The DNA pathogen amplification program was as follows: the first step was 95°C for 30 seconds, the second step, 95°C for 5 seconds, and 60°C for 30 seconds, and the signal acquisition, a total of 40 cycles; RNA pathogen amplification program is: the first step 42°C2 5min, 95°C 30 seconds, the second step, 95°C 5 seconds, 60°C 30 seconds, and signal acquisition, a total of 40 cycles. At the end of amplification, the results were interpreted by fluorescence channels such as FAM, HEX, ROX, CY5, etc. Ct ≤ 36 was judged as positive, 36 < Ct ≤ 38 was weakly positive, and Ct > 38 or no amplification was negative. Positive and quality control were set for each batch, positive quality control Ct ≤ 35, negative no amplification. If the control fails, it needs to be redone.

### 2.5 Statistical analysis

For samples with multiple organisms detected, performance metrics were calculated on a per-sample basis. A sample was considered positive if at least one pathogen matched the composite clinical reference standard. Additional organisms beyond the first were not independently weighted in sensitivity or specificity analyses.

Categorical variables were expressed as counts and percentages and compared using the Chi-square test or Fisher’s exact test, as appropriate. Continuous variables were presented as mean ± standard deviation (SD) if normally distributed, or as median with interquartile range (IQR) otherwise, and compared using the *t*-test or Wilcoxon rank-sum test.

Diagnostic performance metrics, including sensitivity, specificity, positive predictive value (PPV), and negative predictive value (NPV), were calculated using 2 × 2 contingency tables. Variables with a *p*-value < 0.10 in univariate analysis were included in the multivariate model. A two-tailed p-value < 0.05 was considered statistically significant. All analyses were performed using R software (version 4.4).

## 3 Results

### 3.1 Patients and sample characteristics

This study included 410 patients diagnosed with bronchiectasis at Anning First People’s Hospital Affiliated to Kunming University of Science and Technology. The mean age of the cohort was 62.09 ± 14.28 years, 50.49% were female (n = 207), 71.46% were non-smokers (n = 293). The median BMI was 22.10 ± 3.97 kg/m^2^. The majority of patients (58.78%, n = 241) had comorbid chronic pulmonary diseases, including COPD (25.12%, n = 103), asthma (11.95%, n = 49), and other lung diseases (11.22%, n = 46), cardiovascular and cerebrovascular diseases (3.14%, n = 14), malignancy (2.20%, n = 9), immunosuppressive conditions (1.71%, n = 7), metabolic diseases (1.71%, n = 7), and risk of aspiration (1.46%, n = 6). Only 41.22% (n = 169) of patients had simple bronchiectasis. At the time of enrollment, 75.37% (n = 309) had experienced at least one acute exacerbation, and 51.72% (n = 211) had respiratory failure. Cystic bronchiectasis was identified in 46.83% of patients (n = 192), and mucus plugging in 47.07% (n = 193). Oxygen therapy was required in 90.95% of patients (n = 372). The mean BSI was 6.84 ± 4.24. Pulmonary function tests revealed a mean FEV_1_ of 77.00 ± 29.00% and a mean FEV_1_/FVC ratio of 72.00 ± 15.00%. Inflammatory biomarkers showed a mean WBC count of 7.65 ± 3.13 × 10^9^/L, a median neutrophil count of 4.82 × 10^9^/L (IQR 3.42 - 6.62), and a median CRP level of 7.55 mg/L (IQR 1.70 - 29.08) (**Table 1**).

**Table 1:** Differences in clinical features of bronchiectasis based on etiological classification.

Variables	Total (n = 410)	*H. influenzae* (n = 110)	*P. aeruginosa* (n = 61)	Mix (n = 17)	Other (n = 222)	Statistic	*P*-value
Age (years), Mean ± SD	62.09 ± 14.28	59.81 ± 14.76	61.46 ± 12.52	60.88 ± 11.66	63.49 ± 14.59	F = 1.73	0.160
BMI (kg/m²), Mean ± SD	22.10 ± 3.97	22.75 ± 4.19	21.25 ± 3.39	22.44 ± 4.99	21.98 ± 3.89	F = 2.00	0.114
BSI, Mean ± SD	6.84 ± 4.24	5.76 ± 3.61	10.70 ± 4.21	9.21 ± 3.36	6.19 ± 3.98	F = 22.26	**<.001**
FEV_1_ (% predicted) , Mean ± SD	77.00 ± 29.00	82.00 ± 28.	64.00 ± 28.00	68.00 ± 31.00	79.00 ± 29.00	F = 6.20	**<.001**
FEV_1_/FVC (% predicted), Mean ± SD	72.00 ± 15.00	74.00 ± 13.00	67.00 ± 15.00	69.00 ± 17.00	73.00 ± 15.00	F = 2.97	**0.032**
WBC (10^9^/L), Mean ± SD	7.65 ± 3.13	7.87 ± 3.25	9.26 ± 3.33	6.86 ± 2.18	7.16 ± 2.93	F = 8.11	**<.001**
ALC (10^9^/L), Mean ± SD	1.53 ± 0.72	1.64 ± 0.66	1.47 ± 0.81	1.22 ± 0.32	1.51 ± 0.74	F = 2.17	0.091
ANC (10^9^/L), M (Q_1_, Q_3_)	4.82 (3.42, 6.62)	4.87 (3.53,6.79)	6.21 (5.07,9.07)	5.03 (3.29,5.86)	4.29 (3.20,5.96)	χ² = 28.73^#^	**<.001**
AEC (10^9^/L), M (Q_1_, Q_3_)	0.07 (0.03, 0.15)	0.06 (0.03,0.14)	0.05 (0.01,0.11)	0.06 (0.04,0.11)	0.09 (0.03,0.17)	χ² = 6.33^#^	0.096
CRP (mg/L), M (Q_1_, Q_3_)	7.55 (1.70, 29.08)	6.90 (1.62,24.92)	16.94 (5.15,50.65)	8.94 (2.24,25.34)	6.20 (1.30,22.88)	χ² = 13.53^#^	**0.004**
Molecular, n (%)						-	**<.001**
False negative	9 (2.49)	0 (0.00)	3 (5.17)	0 (0.00)	6 (3.35)		
False positive	19 (5.25)	0 (0.00)	1 (1.72)	0 (0.00)	18 (10.06)		
True negative	41 (11.33)	0 (0.00)	0 (0.00)	0 (0.00)	41 (22.91)		
True positive	293 (80.94)	108 (100.00)	54 (93.10)	17 (100.00)	114 (63.69)		
Culture, n (%)						-	**<.001**
False positive	31 (7.62)	9 (8.18)	7 (11.48)	1 (6.25)	14 (6.36)		
False negative	187 (45.95)	79 (71.82)	20 (32.79)	6 (37.50)	82 (37.27)		
True negative	73 (17.94)	0 (0.00)	0 (0.00)	0 (0.00)	73 (33.18)		
True positive	116 (28.50)	22 (20.00)	34 (55.74)	9 (56.25)	51 (23.18)		
Sex, n (%)						χ² = 7.14	0.068
female	207 (50.49)	61 (55.45)	34 (55.74)	12 (70.59)	100 (45.05)		
male	203 (49.51)	49 (44.55)	27 (44.26)	5 (29.41)	122 (54.95)		
Acute Exacerbation, n (%)						χ² = 8.93	**0.030**
no	101 (24.63)	30 (27.27)	14 (22.95)	9 (52.94)	48 (21.62)		
yes	309 (75.37)	80 (72.73)	47 (77.05)	8 (47.06)	174 (78.38)		
Smoking History, n (%)						-	0.630
no	292 (71.39)	83 (75.45)	46 (75.41)	12 (70.59)	151 (68.33)		
previous	4 (0.98)	0 (0.00)	1 (1.64)	0 (0.00)	3 (1.36)		
yes	113 (27.63)	27 (24.55)	14 (22.95)	5 (29.41)	67 (30.32)		
Respiratory Failure, n (%)						χ² = 9.76	**0.021**
no	197 (48.28)	64 (58.18)	21 (34.43)	6 (37.50)	106 (47.96)		
yes	211 (51.72)	46 (41.82)	40 (65.57)	10 (62.50)	115 (52.04)		
Mucus Plug, n (%)						χ² = 7.02	0.071
no	217 (52.93)	54 (49.09)	26 (42.62)	7 (41.18)	130 (58.56)		
yes	193 (47.07)	56 (50.91)	35 (57.38)	10 (58.82)	92 (41.44)		
Cystic Bronchiectasis, n (%)						χ² = 40.47	**<.001**
no	218 (53.17)	76 (69.09)	15 (24.59)	3 (17.65)	124 (55.86)		
yes	192 (46.83)	34 (30.91)	46 (75.41)	14 (82.35)	98 (44.14)		
Oxygen Therapy, n (%)						χ² = 11.82	**0.008**
no	37 (9.05)	17 (15.45)	0 (0.00)	1 (5.88)	19 (8.60)		
yes	372 (90.95)	93 (84.55)	61 (100.00)	16 (94.12)	202 (91.40)		
Underlying disease, n (%)						-	0.223
COPD	103 (25.12)	22 (20.00)	23 (37.70)	7 (41.18)	51 (22.97)		
Asthma	49 (11.95)	9 (8.18)	6 (9.84)	1 (5.88)	33 (14.86)		
other lung diseases	46 (11.22)	16 (14.55)	4 (6.56)	2 (11.76)	24 (10.81)		
Cardiovascular and cerebrovascular diseases	14 (3.41)	4 (3.64)	0 (0.00)	1 (5.88)	9 (4.05)		
Immunosuppressive	7 (1.71)	2 (1.82)	0 (0.00)	0 (0.00)	5 (2.25)		
Malignant tumor	9 (2.20)	3 (2.73)	1 (1.64)	0 (0.00)	5 (2.25)		
Metabolic diseases (diabetes)	7 (1.71)	5 (4.55)	0 (0.00)	0 (0.00)	2 (0.90)		
Risk of aspiration	6 (1.46)	1 (0.91)	0 (0.00)	1 (5.88)	4 (1.80)		
None	169 (41.22)	48 (43.64)	27 (44.26)	5 (29.41)	89 (40.09)		

Based on microbiological classification, patients were grouped into the *Haemophilus influenzae* group, the *Pseudomonas aeruginosa* group, and the mixed infection group (co-infected with *H. influenzae* and *P. aeruginosa*). Infections not belonging to these three categories were classified as “other.” Data are consistently summarized within rows: mean ± standard deviation (SD) is used for normally distributed variables, and median (interquartile range, IQR) is used for non-normally distributed variables. Categorical variables are shown as number (percentage). Statistical comparisons were performed using one-way ANOVA (F), Kruskal–Wallis test (#), Chi-square test (χ²), or Fisher’s exact (–). Bold values indicate statistical significance (p < 0.05). BMI, body mass index; BSI, Bronchiectasis Severity Index; FEV_1_, forced expiratory volume in 1 second; FVC, forced vital capacity; WBC, white blood cell count; ALC, absolute lymphocyte count; ANC, absolute neutrophil count; AEC, absolute eosinophil count; CRP, C-reactive protein.

### 3.2 Distribution of pathogenic species in patients with bronchiectasis

**Figure 1:**
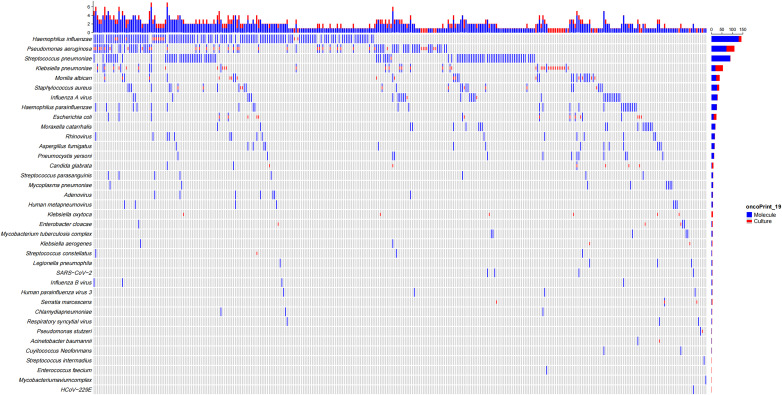
Distribution of pathogens detected by molecular methods and conventional culture in patients with bronchiectasis.

**Figure 1** illustrates the distribution of pathogens detected in patients with bronchiectasis in this study. The most frequently identified bacteria were *H. influenzae* (n = 110, 26.83%), *P. aeruginosa* (n = 61, 14.88%), *Streptococcus pneumoniae* (n = 54, 13.17%), *Klebsiella pneumoniae* (n = 37, 9.02%), *Staphylococcus aureus* (n = 18, 4.39%), *Moraxella catarrhalis* (n = 14, 3.42%), *Escherichia coli* (n = 11, 2.68%), *H. parainfluenzae* (n = 5, 1.22%), and *Mycobacterium tuberculosis* (n = 5, 1.22%). Less frequently detected organisms included non-tuberculous mycobacteria (*Mycobacterium avium* and *Mycobacterium terrae*, one case each), *Streptococcus constellatus* (n = 2), *Nocardia* spp. (n = 1), and *Legionella pneumophila* (n = 1). Among respiratory viruses, the most common were *influenza A virus* (n = 24, 5.85%), rhinovirus (n = 8, 1.95%), SARS-CoV-2 (n = 6, 1.46%), human metapneumovirus (n = 3, 0.73%), influenza B virus (n = 3, 0.73%), and *Respiratory syncytial virus* (n = 2, 0.49%), while parainfluenza virus and adenovirus were each detected in one case. The most commonly detected fungi included *Aspergillus fumigatus* (n = 8, 1.95%) and *Candida albicans* (n = 4, 0.97%), with one case each of *Cryptococcus neoformans* and *Pneumocystis jirovecii*. *Mycoplasma pneumoniae* was identified in 8 cases (1.95%) and *Chlamydia pneumoniae* in 3 cases (0.73%). Mixed infections involving two or more pathogens were observed in 80 patients (19.51%), and no pathogen was detected in 77 patients (18.78%).

Among bronchiectasis patients with comorbid COPD or asthma, the three main pathogens identified were *P. aeruginosa*, *H. influenzae*, and *Streptococcus pneumoniae* (**Supplementary Figure A2**). Notably, *P. aeruginosa* was more frequently detected in patients with comorbid COPD, whereas *H. influenzae* was more commonly found in those with comorbid asthma.

### 3.3 Comparison of molecular detection and traditional pathogen detection

Among 410 patients with bronchiectasis, 361 underwent both CMT and molecular diagnostic testing, including 212 underwent multiplex qPCR testing, 65 underwent targeted tNGS testing, and 84 underwent mNGS testing. The overall detection rate of molecular diagnostic methods was significantly higher than that of CMT (79.78% vs. 32.69%, *p* < 0.001).

**Figure 2:**
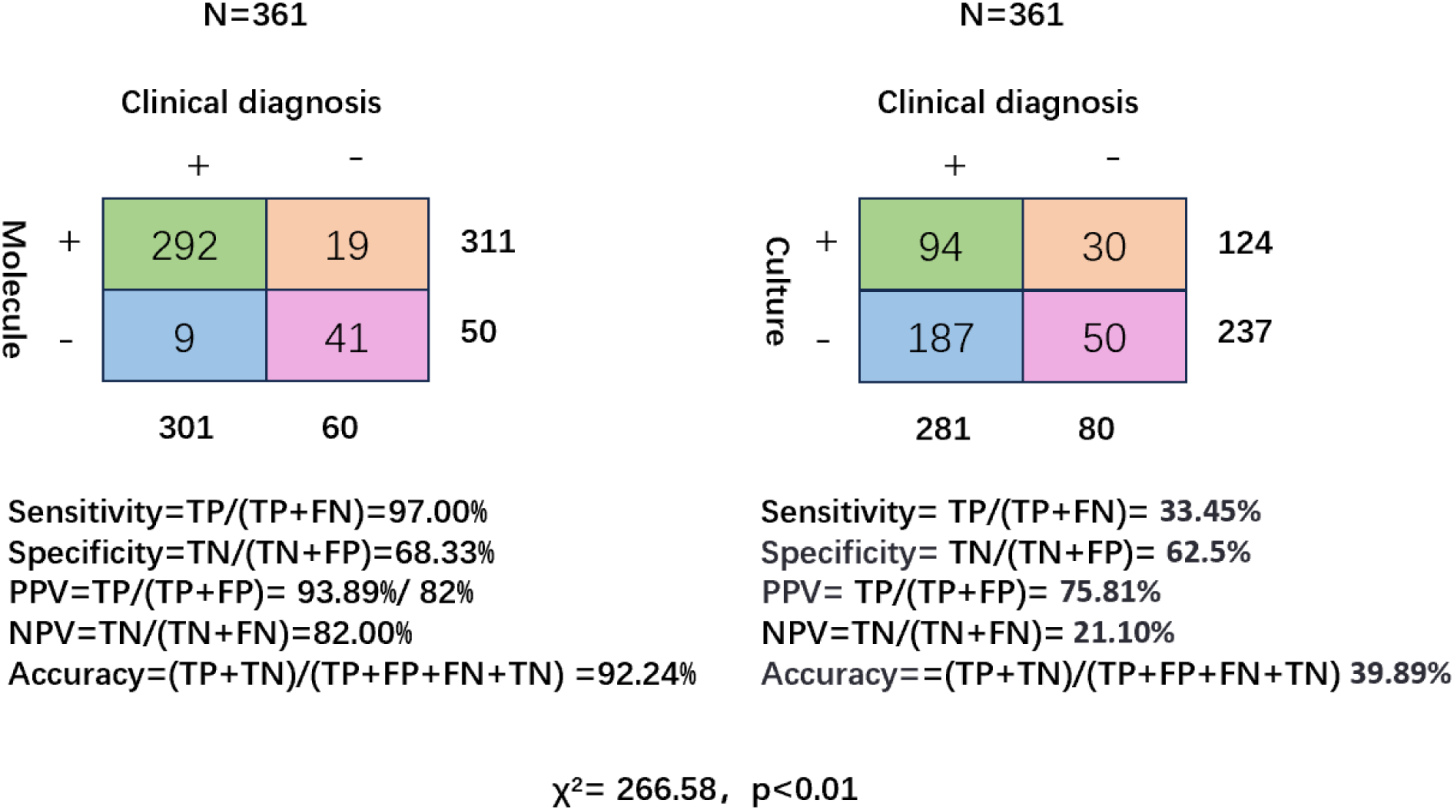
Diagnostic performance of molecular testing and conventional culture in patients with bronchiectasis. Two-by-two contingency tables comparing molecular diagnostics (left) and conventional culture (right) against clinical diagnosis as the reference standard (N = 361). Sensitivity, specificity, positive predictive value (PPV), negative predictive value (NPV), and overall accuracy are shown for each method.

In terms of diagnostic performance, molecular methods demonstrated significantly superior sensitivity compared to conventional culture (97.00% vs. 33.45%, *p* < 0.001, 95% CI: 0.58-0.59), as well as higher specificity (68.33% vs. 62.5%, *p* = 0.47, 95% CI: -0.1-0.22). The PPV of molecular methods was also significantly higher (93.89% vs. 75.81%, *p* < 0.001, 95% CI: 0.1-0.26), along with a markedly greater NPV (82.00% vs. 21.10%, *p* < 0.001, 95% CI: 0.49-0.73). Additionally, the overall diagnostic accuracy of molecular techniques reached 92.24%, significantly exceeding that of conventional culture (39.89%, *p* < 0.001, 95% CI: 0.47-0.58) (**Figure 2**).

In addition to superior diagnostic sensitivity and predictive values, As shown in **Figure 1**, molecular diagnostics identified a broader range of pathogens, including fastidious organisms and mixed infections that were frequently missed by conventional culture, such as Nocardia, Legionella, and Nontuberculous mycobacteria (NTM), and Chlamydia pneumoniae, which are all obtained in molecular testing (especially tNGS and mNGS, which do not include pathogens in qPCR). In addition, the molecular detection rates of *Mycoplasma pneumoniae*, respiratory viruses (such as influenza A virus, rhinovirus, metapneumovirus), and fungal pathogens (such as Aspergillus fumigatus, Candida albicans) were also higher than those of conventional testing. 102 patients (24.88%) were infected with two or more mixed pathogens.

**Figure 3:**
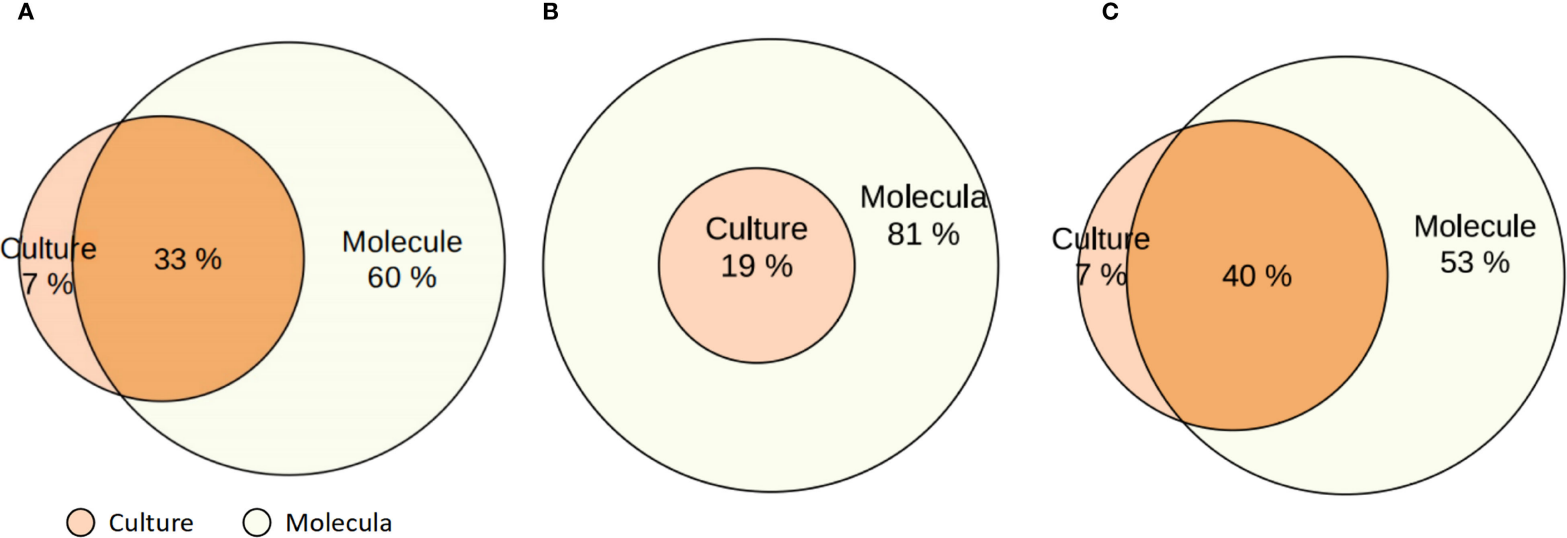
Venn diagrams comparing pathogen detection by molecular methods and conventional culture in patients with bronchiectasis. **(A)** Overall comparison of detection rates by molecular diagnostics and culture across all identified pathogens. **(B)** Detection of *Haemophilus influenzae* by molecular methods and culture. **(C)** Detection of *Pseudomonas aeruginosa* by molecular methods and culture. Percentages indicate the proportion of positive results detected exclusively by culture, exclusively by molecular methods, and by both.

As shown in the Venn diagrams (**Figure 3**), molecular diagnostics uniquely identified 60% of pathogens not detected by culture, while only 7% were detected exclusively by culture, *Escherichia coli, Enterobacter cloacae, Stenotrophomonas maltophilia, Serratia marcescens, Klebsiella oxytoca* are usually only detected in culture; 33% of cases were identified by both methods (**Figure 3A**). *H. influenzae* was more frequently identified by molecular methods (all *H. influenzae* molecular tests were positive, whereas only 19% were culture-positive) (**Figure 3B**), and *P. aeruginosa* was more frequently identified by molecular methods (93% were positive for *H. influenzae*, whereas only 47% were culture-positive), whereas 7% of *P. aeruginosa* molecular tests were false-negative (**Figure 3C**).

Of note, when considering pathogen distribution based solely on culture results, the top five pathogens were *P. aeruginosa* in 37 cases (9.02%), *Klebsiella pneumoniae* in 35 cases (8.54%), *Escherichia coli* in 13 cases (3.17%), *H. influenzae* in 12 cases (2.93%), and *Staphylococcus aureus* in 10 cases (2.44%). In contrast, based exclusively on molecular detection, the top five pathogens were *H. influenzae* in 110 cases (26.83%), *P. aeruginosa* in 57 cases (13.17%), *Streptococcus pneumoniae* in 54 cases (13.17%), influenza A virus in 24 cases (5.85%), and *Klebsiella pneumoniae* in 20 cases (4.88%).

### 3.4 Comparison of clinical characteristics among patients with different pathogens

Patients were categorized into four groups based on etiological classification: *H. influenzae* (n = 110), *P. aeruginosa* (n = 61), mixed infection group co-infected with both *H. influenzae* and *P. aeruginosa* (n = 17), and the “other” group (n = 222) which included all other detected pathogens. The clinical features varied significantly across these groups (**Table 1**).

The mean BSI was significantly higher in the *P. aeruginosa* group (10.70 ± 4.21) compared to the *H. influenzae* group (5.76 ± 3.61), the mixed infection group (9.21 ± 3.36), and the other group (6.19 ± 3.98) (*p* < 0.001). The mean FEV_1_ was 64.00 ± 28.00% in the *P. aeruginosa*, which was significantly lower than in the *H. influenzae* (82.00 ± 28.00%) and the other group (79.00 ± 29.00%) (*p* < 0.001). Similarly, the mean FEV_1_/FVC ratio in the *P. aeruginosa* was 0.67 ± 0.15%, compared to 74.00 ± 13.00% in the *H. influenzae* and 73.00 ± 15.00% in the other group (*p* = 0.032). Inflammatory markers were significantly elevated in the *P. aeruginosa* group compared to the other groups. The mean WBC count in the *P. aeruginosa* was 9.26 ± 3.33 × 10^9^/L, which was higher than in the *H. influenzae* (7.87 ± 3.25 × 10^9^/L) and the other group (7.16 ± 2.93 × 10^9^/L), with *p* < 0.01. Similarly, the median ANC was 6.21 × 10^9^/L (IQR 5.07–9.07) in the *P. aeruginosa*, versus 4.87 × 10^9^/L (IQR 3.53–6.79) in the *H. influenzae* and 4.29 × 10^9^/L (IQR 3.20–5.96) in the other group (*p* < 0.01). The median CRP level was also markedly elevated in the *P. aeruginosa* at 16.94 mg/L (IQR 5.15–50.65), compared with 6.90 mg/L (IQR 1.62–24.92) in the *H. influenzae* and 6.20 mg/L (IQR 1.30–22.88) in the other group (*p* < 0.01).

Cystic bronchiectasis was markedly more prevalent among patients in the *P. aeruginosa* (75.41%) and mixed infection (82.35%) groups compared to the *H. influenzae* (30.91%) and other groups (44.14%) (*p* < 0.001). Furthermore, the *P. aeruginosa* and mixed groups showed higher incidences of respiratory failure, with 65.57% and 62.50% of patients affected, respectively, compared to 41.82% in *H. influenzae* and 52.04% in the other group (*p* = 0.021). The requirement for oxygen therapy was also significantly more frequent in the *P. aeruginosa* (100.00%) and the mixed group (94.12%) compared to the *H. influenzae* (84.55%) and the other group (91.40%) (*p* = 0.008).

We compared the characteristics of patients infected with *P. aeruginosa* and *H. influenzae*, the two most common bacterial pathogens identified. Patients infected with *P. aeruginosa* exhibited significantly worse lung function, as evidenced by lower FEV_1_ (64.00 ± 28.00% vs. 82.00% ± 28.00%, *p* < 0.001) and FEV_1_/FVC ratio (67.00 ± 15.00% vs. 74.00 ± 13.00%, *p* = 0.003). Inflammatory burden was also higher in the *P. aeruginosa* group, with elevated WBC counts (9.26 ± 3.33 × 10^9^/L vs. 7.87 ± 3.25 × 10^9^/L, *p* = 0.009) and CRP levels (42.17 ± 63.91 mg/L vs. 21.68 ± 37.97 mg/L, *p* = 0.026). Radiologically, *P. aeruginosa* was more frequently associated with cystic bronchiectasis (75.41% vs. 30.91%, *p* < 0.001), and clinically, patients with this infection had significantly higher rates of respiratory failure (65.57% vs. 41.82%, *p* = 0.003) and oxygen therapy requirements (100% vs. 84.55%, *p* = 0.001) (**Supplementary Table A2**).

The BSI scores of bronchiectasis patients with comorbid COPD were significantly higher than those of patients with comorbid asthma (**Supplementary Figure A1–A**, *p* < 0.001). Regarding the distribution of BSI severity, patients with COPD were more likely to present with severe disease, whereas those with asthma were more frequently classified as having mild or moderate disease (**Supplementary Figure A1–B**, *p* < 0.01). The FEV_1_ (percent predicted value) (**Supplementary Figure A1–C**, *p* < 0.001) and FEV_1_/FVC ratio (**Supplementary Figure A1–D**, *p* < 0.01) in the bronchiectasis combined with COPD were significantly lower than those in combined asthma.

**Figure 4:**
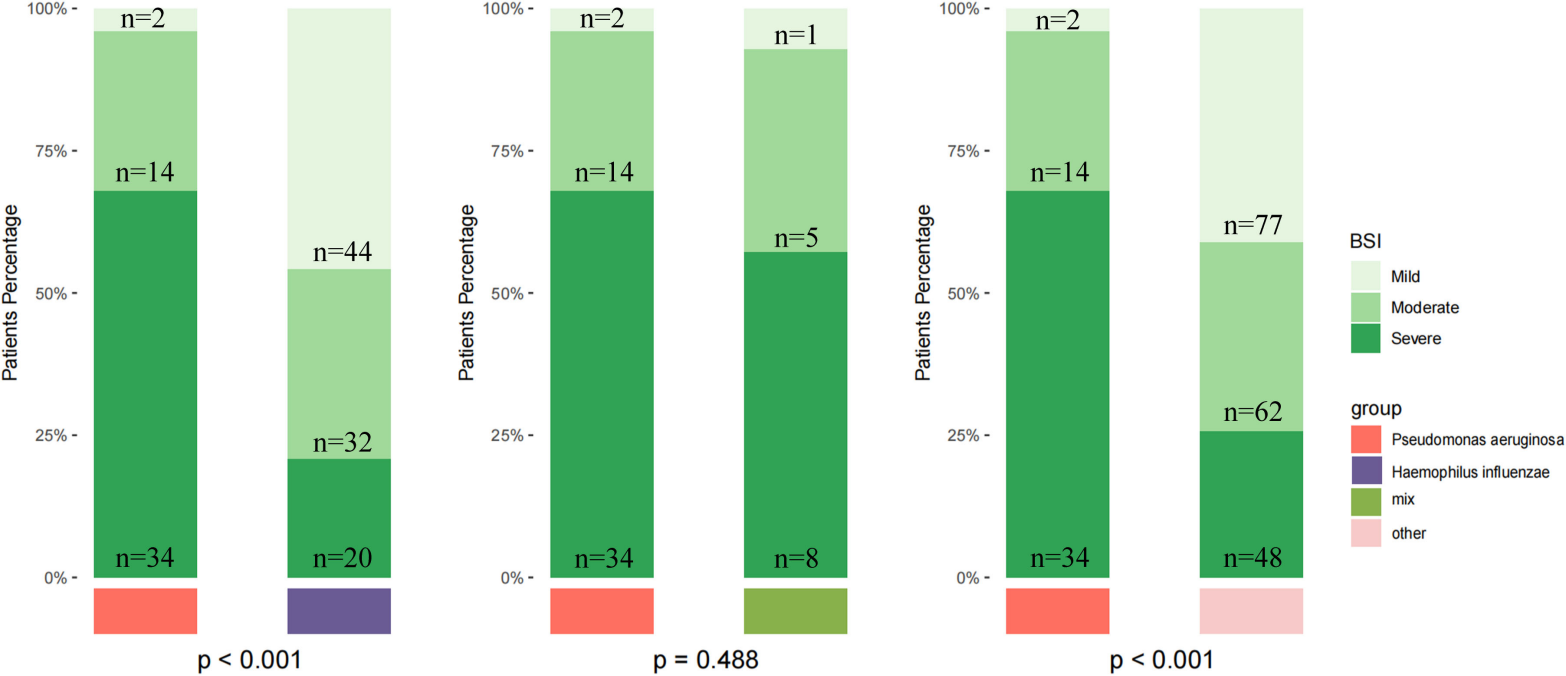
Distribution of bronchiectasis severity by pathogen group based on the Bronchiectasis Severity Index (BSI). Patients were stratified into mild, moderate, and severe categories according to BSI scores. The number of patients in each stratum (n) is indicated. Severity distributions are shown for patients infected with *Pseudomonas aeruginosa*, *Haemophilus influenzae*, those with mixed infections, and other pathogens. Statistical comparisons among groups were performed using the chi-square test, and the reported *p*-values were not adjusted for multiple testing.

Finally, we stratified disease severity based on BSI scores and found significant difference between specific pathogens and bronchiectasis severity (**Figure 4**). *P. aeruginosa* infection is more common in patients with severe bronchiectasis, while *H. influenzae* was more common in those with mild to moderate severity (*p* < 0.001).

### 3.5 Pathogen distribution and diagnostic performance in stable and exacerbation phases

In the stable phase, the most frequently detected bacteria were *H. influenzae*, *P. aeruginos*a, and *Streptococcus pneumoniae*. During acute exacerbations, *P. aeruginosa* and *H. influenzae* remained the leading pathogens, followed by *Klebsiella pneumoniae*. In addition, respiratory viruses such as influenza A/B, rhinovirus, and SARS-CoV-2 were more commonly identified in the exacerbation group, while fungal pathogens including *Aspergillus fumigatus* were occasionally observed in both phases (**Supplementary Figure A3**).

Regarding diagnostic performance, in the stable phase (n = 88), molecular testing achieved a sensitivity of 94.94%, specificity of 44.44%, and accuracy of 89.77%, compared with culture, which showed markedly lower sensitivity (34.21%), specificity (50.00%), and accuracy (36.36%) (χ² = 63.448, *p* < 0.01). During acute exacerbations (n = 273), molecular diagnostics maintained a high sensitivity (97.75%), specificity (72.55%), and accuracy (93.04%), whereas culture yielded substantially lower sensitivity (33.17%), specificity (64.71%), and accuracy (41.03%) (χ² = 203.84, *p* < 0.01) (**Supplementary Figure A4**). Overall, the sensitivity, specificity, positive predictive value, and negative predictive value of molecular testing were significantly higher than those of conventional methods.

## 4 Discussion

This study is the largest retrospective single-center cohort study of adults with bronchiectasis in Yunnan, China to date. Our findings reveal significant advantages of molecular diagnostics over conventional culture methods, demonstrating markedly higher sensitivity, positive predictive value, and negative predictive value. Importantly, our study identified that the pathogen spectrum in Yunnan bronchiectasis patients was predominantly characterized by *H. influenzae* as the most frequently detected pathogen, followed by *P. aeruginosa*. Molecular methods further broadened the pathogen spectrum, enabling precise identification of fastidious and uncommon organisms often undetected by culture. Moreover, we observed distinct clinical phenotypes associated with different pathogens; patients infected with *P. aeruginosa* exhibited more severe disease manifestations, including poorer lung function, higher inflammatory markers, and greater requirements for oxygen therapy compared to those infected with *H. influenzae*.

Our findings are consistent with previous reports indicating the superior sensitivity and diagnostic performance of molecular methods over traditional culture techniques in respiratory infections (Pandey et al., 2022; Gao et al., 2024; Chen et al., 2025; Ding et al., 2025). Specifically, molecular diagnostics in our cohort achieved significantly higher sensitivity (96.97% vs. 33.45%, *p* < 0.001), specificity (68.33% vs. 62.5%, *p* = 0.039), positive predictive value (93.89% vs. 75.81%, *p* < 0.001), and negative predictive value (82.00% vs. 21.10%, *p* < 0.001) compared to CMT.

Subgroup analysis showed that molecular methods consistently outperformed culture in both stable and exacerbation phases. In the stable phase, molecular testing achieved high sensitivity (94.94%) but relatively low specificity (44.44%), which may be explained by the relatively small sample size, and the fact that many patients were not experiencing active infection. Moreover, our study included different molecular platforms (mNGS, tNGS, and especially multiplex qPCR), and variability in target design and reporting thresholds may have contributed to false-positive results. In contrast, during acute exacerbations, molecular diagnostics maintained excellent sensitivity (97.75%) and substantially higher specificity (72.55%) than in the stable phase, whereas culture remained markedly inferior (sensitivity 33.2%, specificity 64.71%). These findings align with recent studies showing that molecular assays provide superior diagnostic yield, particularly in acute disease when pathogen burden and clinical relevance are greater (Candel et al., 2024; Liu et al., 2024; Jiang et al., 2025). Overall, molecular diagnostics offer clear advantages over culture, but results in the stable phase should be interpreted with clinical (Baghdadi et al., 2023; De Angelis et al., 2025).

Geographical and socioeconomic factors contribute to substantial variations in the pathogen spectrum of bronchiectasis. The EMBARC multicenter cohort study from Europe and the BE-China multicenter cohort study from China showed that *P. aeruginosa* was the most commonly isolated bacteria in patients with bronchiectasis (Chalmers et al., 2023; Xu et al., 2025). Many studies from different regions have emphasized the important pathogenic role of *P. aeruginosa* in bronchiectasis, and the research on this pathogen has received the most attention (Jin et al., 2025; Sun et al., 2025; Wen et al., 2025). Subgroup analysis of the EMBARC cohort study from Europe showed that *H. influenzae* was more common in the United Kingdom and northern and western Europe, which is similar to our findings, with *H. influenzae* infection being the most common infection in our cohort. Compared with infections caused by *P. aeruginosa*, bronchiectasis associated with *H. influenzae* tended to present with milder symptoms, less severe disease, and slower progression. Therefore, research on *H. influenzae* in bronchiectasis remains limited (Yang et al., 2024).

In bronchiectasis, *H. influenzae* is widely recognized as one of the major bacterial pathogens rather than a mere colonizer, as emphasized by both European and Chinese expert consensus statements (Polverino et al., 2017; Chalmers et al., 2023; China Bronchiectasis Alliance (BE-China) et al., 2024). The clinical relevance of its detection in our study is further supported by the fact that positive molecular findings were accompanied by compatible symptoms, radiological abnormalities, and clinical improvement following targeted antimicrobial therapy. These observations suggest that the majority of *H. influenzae* detections reflected true infection rather than transient colonization or residual nucleic acids from non-viable organisms.

The culture positivity rate for *P. aeruginosa* was relatively high, but molecular diagnostics still significantly increased the overall positive detection rate of these pathogens. In contrast, the culture of *H. influenzae* had a high false-negative rate, while molecular methods achieved a true-positive rate of up to 100%. This difference arose because *H. influenzae* was a fastidious organism that required stringent culture conditions, often leading to missed or misdiagnosed cases in clinical practice (King, 2012; Mojebi et al., 2024; Do Carmo Silva et al., 2025). Therefore, molecular methods demonstrated clear advantages in the detection and identification of fastidious pathogens such as *H. influenzae*.

Respiratory viruses, including influenza A/B, rhinovirus, and SARS-CoV-2, were more frequently identified during exacerbations, underscoring their recognized role as triggers of AEs. Previous studies have demonstrated that respiratory viruses, especially influenza and rhinoviruses, account for a considerable proportion of exacerbations, and reductions in viral circulation during the COVID-19 pandemic were paralleled by declines in bronchiectasis exacerbations (Crichton et al., 2021; Huang et al., 2023; Morelli et al., 2025). These observations support the clinical importance of incorporating molecular viral diagnostics in AE evaluations, particularly during seasonal peaks, and suggest potential benefits of antiviral or vaccination strategies.

The results from our center showed a lower detection rate of tuberculosis-associated bronchiectasis compared to the BE-China study (5/410, 1.22% vs. 98/3892, 2.6%), the proportion of post-infectious causes and tuberculosis-related bronchiectasis in Chinese cohorts was higher than that reported in the EMBARC cohort. We believe this is attributable to China being a country with a high tuberculosis burden, where a considerable proportion of bronchiectasis cases are secondary to old tuberculosis lesions, resulting in a heavier disease burden and a lower proportion of current infections. In the United States, Japan, South Korea, and China’s coastal economically developed areas, NTM have been detected at relatively high rates among patients with bronchiectasis (Park et al., 2021; Aksamit et al., 2025; Fujita, 2025; Zhang et al., 2025). However, in our study, the detection rate of NTM was low, with only two cases identified. Similarly, in the BE-China study, Aspergillus fumigatus ranked eighth in detection frequency (194/4395, 4.4%), whereas in our cohort, the detection rate was only 1.95% (8/410). This discrepancy may be related to the high-altitude environment, dry climate, local socioeconomic conditions, and environmental exposures in Yunnan.

In Yunnan, China, apart from a few specialized infectious disease hospitals, the vast majority of general hospitals are unable to perform isolation and identification of *tuberculosis* and NTM. Therefore, relying solely on traditional microbiological methods to diagnose these infections is extremely challenging. The same problem exists in our center, conventional microbiological tests have little diagnostic value for these rare or chronic pathogens. Final diagnoses are predominantly based on molecular detection methods, combined with clinical manifestations and follow-up after treatment (Wang et al., 2022; Liu et al., 2023). We therefore believe that molecular diagnostics offer significant advantages in the detection and identification of rare pathogens associated with bronchiectasis.

From a clinical perspective, accurate and timely pathogen identification is essential in bronchiectasis, given the heterogeneity of the disease and the role of infections in driving exacerbations and lung damage. Our results suggest that molecular diagnostics can not only improve microbiological diagnosis rates but also support individualized treatment decisions, potentially guiding targeted antimicrobial therapy and reducing unreasonable use of broad-spectrum antibiotics.

This study observed that chronic airway diseases often coexist with bronchiectasis, with 25.12% of patients having COPD and 11.95% having asthma. This highlights the complex interaction between chronic airway diseases and the development and progression of bronchiectasis. Recent evidence by He et al. (2025) has highlighted that patients with COPD-bronchiectasis overlap exhibit distinct sputum microbiota compositions and metabolomic profiles, which correlate with worse clinical outcomes and frequent exacerbations. Their findings suggest that the coexistence of COPD not only predisposes patients to structural airway damage characteristic of bronchiectasis but also contributes to a unique inflammatory and microbial environment that may exacerbate disease severity (Zhang et al., 2025). We further compared pulmonary function and BSI) between patients with bronchiectasis combined with COPD and those with bronchiectasis combined with asthma. The results showed that patients with coexisting COPD had significantly higher BSI scores, more severe disease classification, and more pronounced impairment in lung functions, specifically, lower FEV_1_% predicted, FEV_1_/FVC and higher rates of *P. aeruginosa* infection—compared to those with coexisting asthma. These findings suggest that COPD as a comorbidity may aggravate the progression of bronchiectasis by accelerating airflow limitation, promoting systemic inflammation, and leading to more frequent exacerbations, high antibiotic burden and hospitalizations (Alam et al., 2024; Chen et al., 2024; Dai et al., 2024). Our data are consistent with these observations and suggest that chronic airway disease may be both a risk factor and a synergistic factor for bronchiectasis, necessitating a more personalized approach to diagnosis and treatment.

Moreover, elevated inflammatory biomarkers in our patients—including increased white blood cell counts, higher neutrophil counts, and elevated C-reactive protein levels—further support the presence of persistent airway inflammation, a hallmark of bronchiectasis pathophysiology. Importantly, neutrophilic inflammation is not merely a passive bystander but actively contributes to tissue injury through mechanisms such as the formation of neutrophil extracellular traps (NETs) (Wigerblad and Kaplan, 2023; King and Dousha, 2024). Recent studies have demonstrated that NETs, while part of the host defense mechanism, can damage the airway epithelium, promote mucus hypersecretion, and perpetuate the vicious cycle of infection and inflammation in bronchiectasis (Keir et al., 2021; Li et al., 2022). This persistent inflammatory milieu may underlie the high rates of exacerbations and the severe clinical phenotype observed in our cohort, particularly among those with COPD or other chronic airway diseases. In summary, these findings emphasize the key role of chronic airway inflammatory diseases in influencing the clinical course and prognosis of bronchiectasis.

This study observed significant differences in clinical characteristics between patients infected with *P. aeruginosa* and those with *H. influenzae*. Specifically, *P. aeruginosa* infection was associated with a lower BMI (21.25 ± 3.39 vs. 22.75 ± 4.19), poorer lung function as reflected by reduced FEV_1_ (64.00 ± 28.00% vs. 82.00 ± 28.00%) and FEV_1_/FVC ratio (67.00 ± 15.00% vs. 74.00 ± 13.00%), and higher levels of systemic inflammation, including elevated WBC counts (9.26 ± 3.33 × 10^9^/L vs. 7.87 ± 3.25 × 10^9^/L) and CRP levels (42.17 ± 63.91 mg/L vs. 21.68 ± 37.97 mg/L). Clinically, patients with *P. aeruginosa* were more likely to develop cystic bronchiectasis, respiratory failure, and require oxygen therapy. These findings are biologically plausible given the pathogen’s capacity to form biofilms, sustain chronic colonization, and secrete diverse virulence factors that perpetuate neutrophilic inflammation and airway remodeling (Malhotra et al., 2019; Fernández-Barat et al., 2025). The systemic spillover of inflammatory mediators may contribute to protein catabolism and malnutrition, providing a mechanistic explanation for the reduced BMI observed in this group. WBC and CRP levels further reflect the inflammatory burden, consistent with previous evidence linking systemic inflammation to disease severity and poor prognosis in bronchiectasis (Keir et al., 2021; Choi et al., 2024). Moreover, chronic infection and frequent multidrug resistance in *P. aeruginosa* facilitate recurrent exacerbations and progressive lung injury, thereby predisposing patients to severe outcomes such as respiratory failure and oxygen dependence (Holgersen et al., 2025; Sun et al., 2025). These findings suggest that P. aeruginosa infection in bronchiectasis is not only associated with greater disease severity but also with more rapid progression, underscoring the need for earlier and more comprehensive interventions to slow disease advancement and mitigate long-term risks.

We also found significant differences in clinical characteristics between patients infected with *P. aeruginosa* and those with *H. influenzae*. Patients infected with P. aeruginosa had a lower BMI (21.25 ± 3.39 vs. 22.75 ± 4.19), poorer lung function as reflected by reduced FEV_1_ (64.00 ± 28.00% vs. 82.00 ± 28.00%) and FEV_1_/FVC ratio (67.00 ± 15.00% vs. 74.00 ± 13.00%), and higher levels of inflammatory markers, including elevated WBC counts (9.26 ± 3.33 × 10^9^/L vs. 7.87 ± 3.25 × 10^9^/L) and CRP levels (42.17 ± 63.91 mg/L vs. 21.68 ± 37.97 mg/L), compared to those infected with H. influenzae. They were more likely to present with cystic bronchiectasis, respiratory failure, and a need for oxygen therapy. These findings are in line with previous studies that identified *P. aeruginosa* as a pathogen associated with more severe disease, increased exacerbations, and worse prognosis in bronchiectasis.

Our study demonstrated a significant association between pathogen type and bronchiectasis severity. *P. aeruginosa* infections were predominantly observed in patients with severe BSI scores, whereas *H. influenzae* was more commonly found in patients with mild to moderate disease. These observations highlight the potential value of pathogen-specific profiling in risk stratification and management of bronchiectasis.

This study has several limitations. First, the retrospective design and single-center setting may limit the generalizability of our findings. We acknowledge that antibiotic exposure and procedural timing can influence diagnostic yield, particularly for culture-based methods. However, due to the retrospective nature of this study, it was not possible to systematically document or exclude patients with antibiotic exposure within 14 days prior to sampling. Future prospective studies should incorporate detailed recording of antibiotic use and standardized timing of sampling to minimize these confounding effects. Second, although molecular diagnosis has significantly improved the detection rate of pathogens, relying solely on molecular testing cannot reliably distinguish colonization from true infection, and requires close combination of clinical and post-treatment follow-up to determine. Third, the absence of long-term follow-up data prevents assessment of how molecular diagnostic results might influence treatment outcomes, disease progression, and prognosis. Furthermore, future studies are warranted to explore the differences and correlations in etiology and clinical characteristics between acute exacerbation and stable phases of bronchiectasis, which could provide deeper insights into disease management and individualized therapy.

## 5 Conclusion

In summary, this study highlights the significant advantages of molecular diagnostic technology over conventional culture technology in the detection of bronchiectasis pathogens. Molecular diagnosis can not only improve sensitivity and diagnostic efficiency, broaden the spectrum of detectable pathogens, reveal pathogen-specific clinical phenotypes associated with disease severity, but also accurately guide clinical anti-infective treatment. There is reason to predict that molecular diagnostic methods have broad application prospects in guiding accurate pathogen diagnosis, personalized treatment, and risk stratification in the management of bronchiectasis.

## Data Availability

The original contributions presented in the study are included in the article/**Supplementary Material**. Further inquiries can be directed to the corresponding authors.
