# Supplementary material for: Precise pathogen detection and clinical characterization of bronchiectasis
Source: Front Cell Infect Microbiol. 2025 Sep 26;15:1670925. doi: 10.3389/fcimb.2025.1670925 (PMC12511037; doi:10.3389/fcimb.2025.1670925)
Supplement: Supplementary file 1 [file Table1.docx]

**Supplementary appendix**

1. Supplementary methods

**Table A1：Target pathogens included in the multiplex qPCR panel.**

The assay simultaneously detects a comprehensive spectrum of respiratory pathogens, including 19 viruses, 13 bacteria, 2 fungi, and 2 atypical pathogens. All bacterial and fungal names are presented in italics according to scientific nomenclature.

| Viruses | Adenovirus |
| --- | --- |
|  | Respiratory syncytial virus, RSV |
|  | Influenza A virus |
|  | Influenza B virus |
|  | Enterovirus spp. |
|  | Rhinovirus spp. |
|  | Human metapneumovirus |
|  | Human gammaherpesvirus 4, EBV |
|  | Human betaherpesvirus 5, HCMV |
|  | Human parainfluenza virus 1 |
|  | Human parainfluenza virus 3 |
|  | Human coronavirus NL63 |
|  | Human coronavirus OC43 |
|  | Human coronavirus 229E |
|  | Human coronavirus HKU1 |
|  | Human bocaparvovirus |
|  | Herpes simplex virus type 1，HSV-1 |
|  | Human herpesvirus 6，HHV-6 |
|  | Human herpesvirus 7，HHV-7 |
| Bacteria | *Mycobacterium tuberculosis* |
|  | *Haemophilus influenzae* |
|  | *Pseudomonas aeruginosa* |
|  | *Klebsiella pneumoniae* |
|  | *Bordetella pertussis* |
|  | *Streptococcus pneumoniae* |
|  | *Legionella pneumophila* |
|  | *Moraxella catarrhalis* |
|  | *Escherichia coli* |
|  | *Acinetobacter baumannii* |
|  | *Streptococcus agalactiae* |
|  | *Staphylococcus aureus* |
|  | *Streptococcus pyogenes* |
| Fungi | *Candida albicans* |
|  | *Aspergillus* spp. |
| Atypical pathogens | *Mycoplasma pneumoniae* |
|  | *Chlamydia pneumoniae* |

1. Supplementary results

**Table A2：Differences in clinical characteristics between the *P.aeruginosa* and the *H.influenzae*.**

| Variables | Total (n = 171) | *P.aeruginosa* (n = 61) | *H.influenzae* (n = 110) | Statistic | *p*-value |
| --- | --- | --- | --- | --- | --- |
|  |  |  |  |  |  |
| Age (years), Mean ± SD | 60.40 ± 13.98 | 61.46 ± 12.52 | 59.81 ± 14.76 | t = 0.74 | 0.461 |
| BMI (kg/m²), Mean ± SD | 22.23 ± 3.98 | 21.25 ± 3.39 | 22.75 ± 4.19 | t = -2.36 | **0.020** |
| FEV_1_ (% predicted)  , Mean ± SD | 76.00 ± 29.00 | 64.00 ± 28.00 | 82.00 ± 28.00 | t = -4.09 | **< .001** |
| FEV_1_ / FVC (% predicted), Mean ± SD | 72.00 ± 14.00 | 67.00 ± 15.00 | 74.00 ± 13.00 | t = -3.06 | **0.003** |
| WBC (10⁹/L), Mean ± SD | 8.37 ± 3.33 | 9.26 ± 3.33 | 7.87 ± 3.25 | t = 2.65 | **0.009** |
| ANC (10⁹/L), Mean ± SD | 6.54 ± 6.99 | 7.04 ± 3.15 | 6.26 ± 8.41 | t = 0.70 | 0.485 |
| ALC (10⁹/L), Mean ± SD | 1.58 ± 0.72 | 1.47 ± 0.81 | 1.64 ± 0.66 | t = -1.49 | 0.139 |
| ANC (10⁹/L), M (Q₁, Q₃) | 0.10 ± 0.11 | 0.10 ± 0.13 | 0.09 ± 0.09 | t = 0.42 | 0.671 |
| AEC (10⁹/L), M (Q₁, Q₃) | 29.00 ± 49.63 | 42.17 ± 63.91 | 21.68 ± 37.97 | t = 2.27 | **0.026** |
| CRP (mg/L), M (Q₁, Q₃) |  |  |  | χ² = 26.08 | **< .001** |
| False negative | 99 (57.89) | 20 (32.79) | 79 (71.82) |  |  |
| False positive | 16 (9.36) | 7 (11.48) | 9 (8.18) |  |  |
| True positive | 56 (32.75) | 34 (55.74) | 22 (20.00) |  |  |
| Molecular, n(%) |  |  |  | - | **0.014** |
| False negative | 3 (1.81) | 3 (5.17) | 0 (0.00) |  |  |
| False positive | 1 (0.60) | 1 (1.72) | 0 (0.00) |  |  |
| True positive | 162 (97.59) | 54 (93.10) | 108 (100.00) |  |  |
| Sex, n(%) |  |  |  | χ² = 0.00 | 0.972 |
| female | 95 (55.56) | 34 (55.74) | 61 (55.45) |  |  |
| male | 76 (44.44) | 27 (44.26) | 49 (44.55) |  |  |
| Acute Exacerbation, n (%) |  |  |  | χ² = 0.38 | 0.536 |
| no | 44 (25.73) | 14 (22.95) | 30 (27.27) |  |  |
| yes | 127 (74.27) | 47 (77.05) | 80 (72.73) |  |  |
| Smoking History, n (%) |  |  |  | - | 0.526 |
| no | 129 (75.44) | 46 (75.41) | 83 (75.45) |  |  |
| previous | 1 (0.58) | 1 (1.64) | 0 (0.00) |  |  |
| yes | 41 (23.98) | 14 (22.95) | 27 (24.55) |  |  |
| Respiratory Failure, n (%) |  |  |  | χ² = 8.86 | **0.003** |
| no | 85 (49.71) | 21 (34.43) | 64 (58.18) |  |  |
| yes | 86 (50.29) | 40 (65.57) | 46 (41.82) |  |  |
| Mucus Plug, n (%) |  |  |  | χ² = 0.66 | 0.417 |
| no | 80 (46.78) | 26 (42.62) | 54 (49.09) |  |  |
| yes | 91 (53.22) | 35 (57.38) | 56 (50.91) |  |  |
| Cystic Bronchiectasis, n (%) |  |  |  | χ² = 31.21 | **< .001** |
| no | 91 (53.22) | 15 (24.59) | 76 (69.09) |  |  |
| yes | 80 (46.78 | 46 (75.41) | 34 (30.91) |  |  |
| Oxygen Therapy, n (%) |  |  |  | χ² = 10.47 | **0.001** |
| no | 17 (9.94) | 0 (0.00) | 17 (15.45) |  |  |
| yes | 154 (90.06) | 61 (100.00) | 93 (84.55) |  |  |
| Underlying disease, n (%) |  |  |  | - | 0.872 |
| Cardiovascular and cerebrovascular diseases | 3 (1.75) | 0 (0.00) | 3 (2.73) |  |  |
| Chronic lung diseases | 105 (61.40) | 40 (65.57) | 65 (59.09) |  |  |
| Comorbidity with multiple underlying diseases | 18 (10.53) | 6 (9.84) | 12 (10.91) |  |  |
| Immunosuppressive | 2 (1.17) | 0 (0.00) | 2 (1.82) |  |  |
| Malignant tumour | 4 (2.34) | 1 (1.64) | 3 (2.73) |  |  |
| Metabolic diseases (diabetes) | 2 (1.17) | 0 (0.00) | 2 (1.82) |  |  |
| none | 36 (21.05) | 14 (22.95) | 22 (20.00) |  |  |
| Risk of aspiration | 1 (0.58) | 0 (0.00) | 1 (0.91) |  |  |

Continuous variables are presented as mean ± standard deviation and compared using Student’s t-test. Categorical variables are presented as numbers and percentages and analyzed using the Chi-square test or Fisher’s exact test where appropriate. Abbreviations: BMI, body mass index; FEV1, forced expiratory volume in 1 second; FVC, forced vital capacity; WBC, white blood cell count; ANC, absolute neutrophil count; ALC, absolute lymphocyte count; AEC, absolute eosinophil count; CRP, C-reactive protein; SD, standard deviation; t, t-test; χ², Chi-square test, -: Fisher exact.


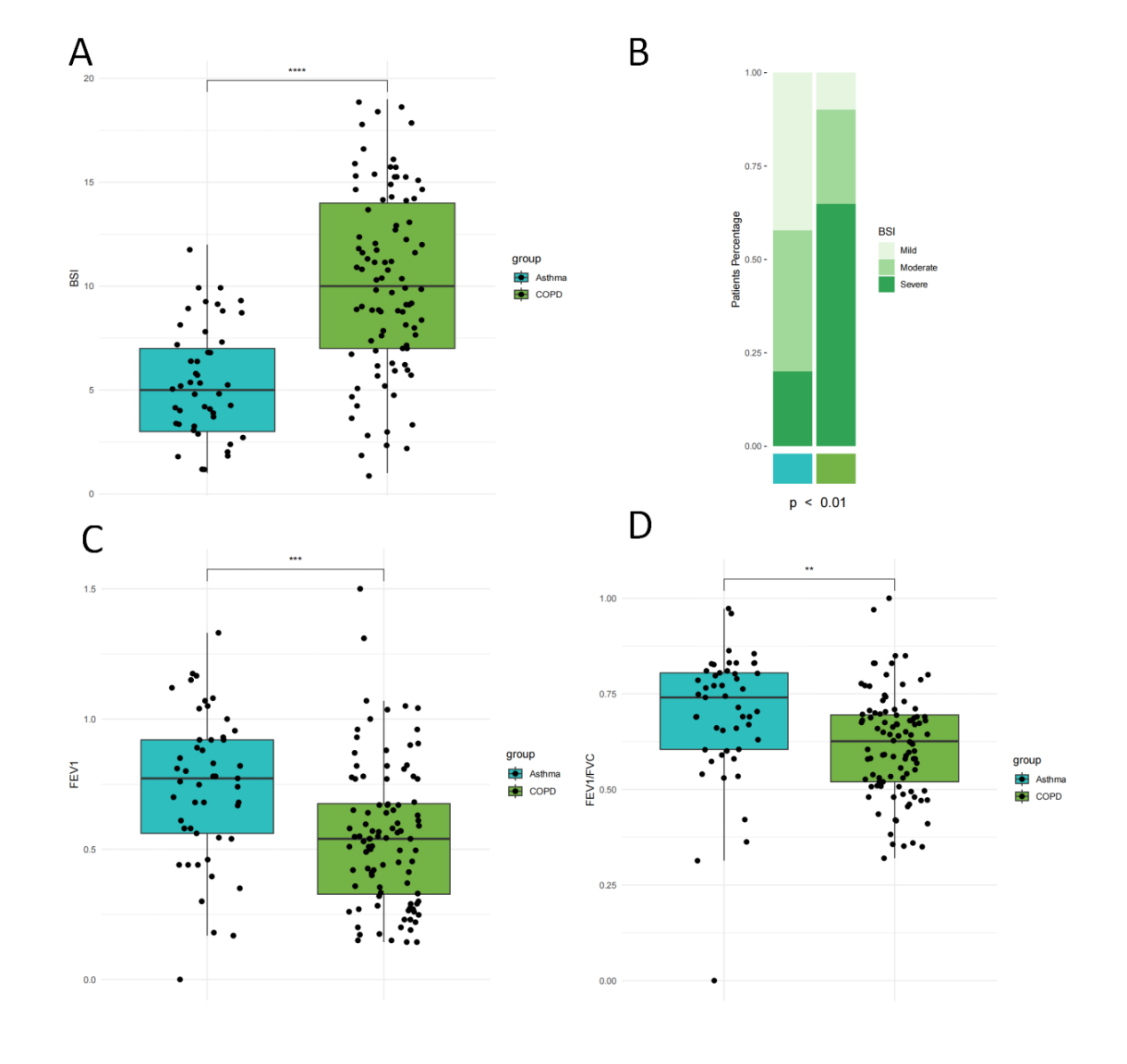


**Figure A1: Figure. Comparison of BSI scores and pulmonary function between bronchiectasis patients with COPD and with asthma.**

A. Comparison of total Bronchiectasis Severity Index (BSI) scores between the BE+COPD and BE+Asthma groups (without severity stratification); B. Distribution of BSI severity grades (mild, moderate, severe) between the two groups; C. Comparison of FEV_1_ (% predicted) between the two groups; D. Comparison of FEV_1_/FVC (%) between the two groups. “**” indicates *P* < 0.01, “***” indicates *P* < 0.001.


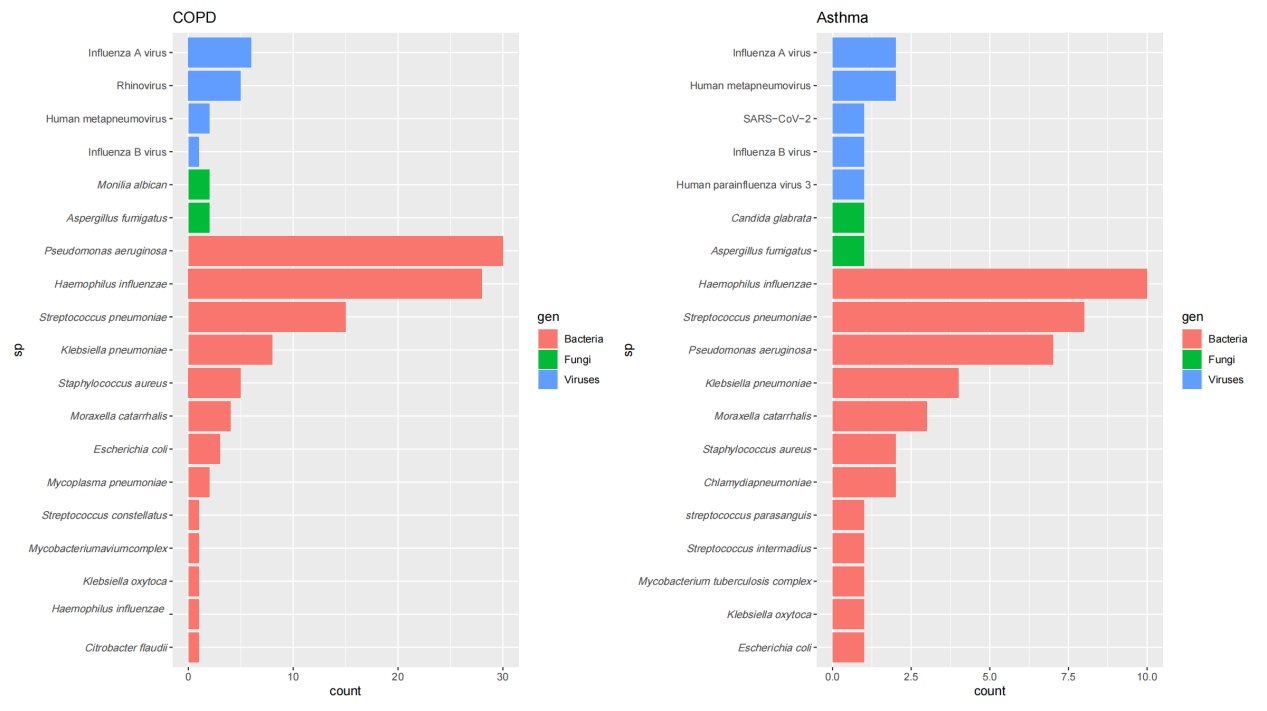


**Figure A2: Comparison of pathogen spectrum between bronchiectasis patients with COPD and with asthma.**


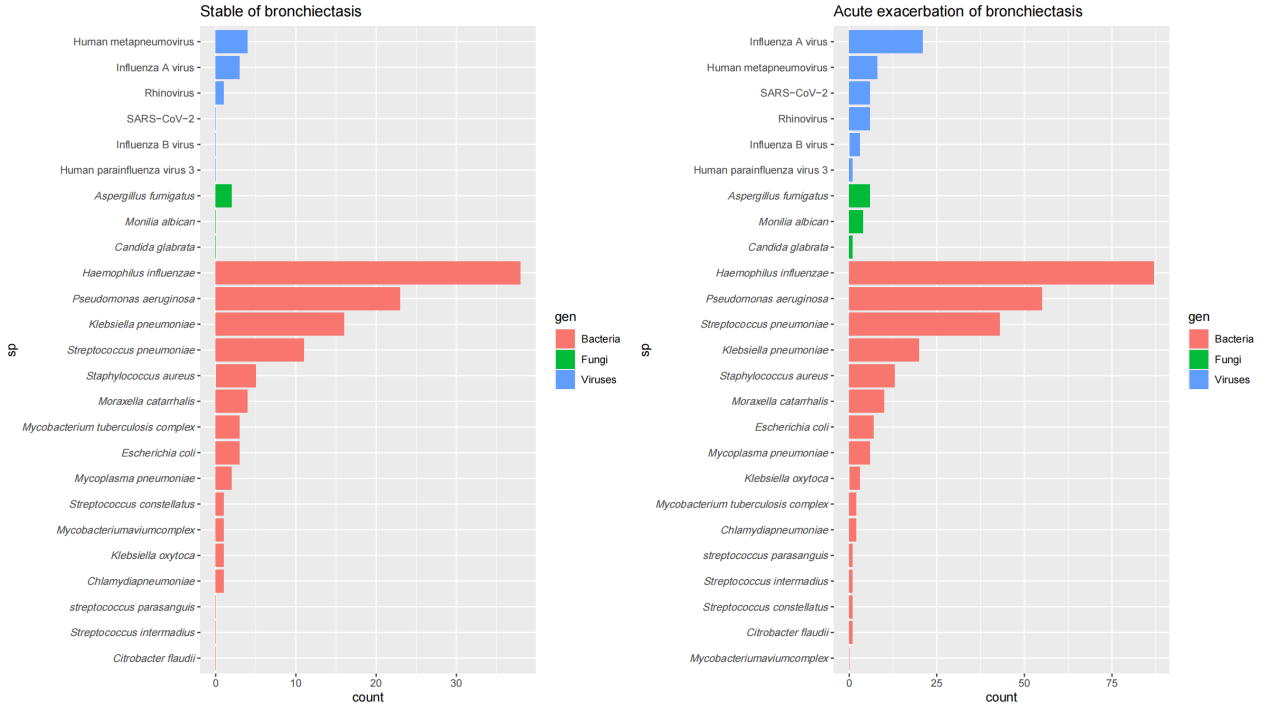


**Figure A3: Distribution of pathogens in patients with bronchiectasis during the stable phase and acute exacerbations.**


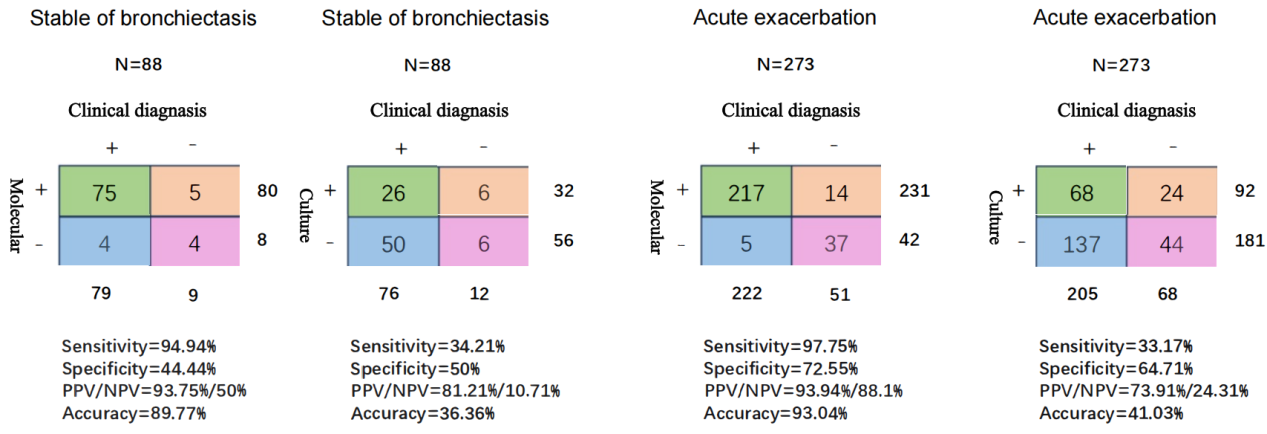


**Figure A4. Diagnostic performance of molecular testing and conventional culture in patients with bronchiectasis during stable phase and acute exacerbations.**

2 × 2 contingency tables comparing molecular diagnostics and conventional culture against clinical diagnosis as the reference standard. The two panels on the left represent patients in the stable phase (N = 88), while the two panels on the right represent patients during acute exacerbations (N = 273). Sensitivity, specificity, positive predictive value (PPV), negative predictive value (NPV), and overall accuracy are shown for each method.
